# Distribution and Clinical Manifestations of *Cryptosporidium* Species and Subtypes in HIV/AIDS Patients in Ethiopia

**DOI:** 10.1371/journal.pntd.0002831

**Published:** 2014-04-17

**Authors:** Haileeyesus Adamu, Beyene Petros, Guoqing Zhang, Hailu Kassa, Said Amer, Jianbin Ye, Yaoyu Feng, Lihua Xiao

**Affiliations:** 1 Division of Foodborne, Waterborne and Environmental Diseases, Centers for Disease Control and Prevention, Atlanta, Georgia, United States of America; 2 Addis Ababa University, Addis Ababa, Ethiopia; 3 Division of Global HIV/AIDS, Centers for Disease Control and Prevention, Atlanta, Georgia, United States of America; 4 Department of Public and Allied Health, Bowling Green State University (BGSU), Bowling Green, Ohio, United States of America; 5 Department of Zoology, Faculty of Science, Kafr El sheikh University, Kafe El Sheikh, Egypt; 6 State Key Laboratory of Bioreactor Engineering, School of Resources and Environmental Engineering, East China University of Science and Technology, Shanghai, People's Republic of China; University of Minnesota, United States of America

## Abstract

**Background:**

Cryptosporidiosis is an important cause for chronic diarrhea and death in HIV/AIDS patients. Among common *Cryptosporidium* species in humans, *C. parvum* is responsible for most zoonotic infections in industrialized nations. Nevertheless, the clinical significance of *C. parvum* and role of zoonotic transmission in cryptosporidiosis epidemiology in developing countries remain unclear.

**Methodology/Principal Findings:**

In this cross-sectional study, 520 HIV/AIDS patients were examined for *Cryptosporidium* presence in stool samples using genotyping and subtyping techniques. Altogether, 140 (26.9%) patients were positive for *Cryptosporidium* spp. by PCR-RFLP analysis of the small subunit rRNA gene, belonging to *C. parvum* (92 patients), *C. hominis* (25 patients), *C. viatorum* (10 patients), *C. felis* (5 patients), *C. meleagridis* (3 patients), *C. canis* (2 patients), *C. xiaoi* (2 patients), and mixture of *C. parvum* and *C. hominis* (1 patient). Sequence analyses of the 60 kDa glycoprotein gene revealed a high genetic diversity within the 82 *C. parvum* and 19 *C. hominis* specimens subtyped, including *C. parvum* zoonotic subtype families IIa (71) and IId (5) and anthroponotic subtype families IIc (2), IIb (1), IIe (1) and If-like (2), and *C. hominis* subtype families Id (13), Ie (5), and Ib (1). Overall, *Cryptosporidium* infection was associated with the occurrence of diarrhea and vomiting. Diarrhea was attributable mostly to *C. parvum* subtype family IIa and *C. hominis*, whereas vomiting was largely attributable to *C. hominis* and rare *Cryptosporidium* species. Calf contact was identified as a significant risk factor for infection with *Cryptosporidium* spp., especially *C. parvum* subtype family IIa.

**Conclusions/Significance:**

Results of the study indicate that *C. parvum* is a major cause of cryptosporidiosis in HIV-positive patients and zoonotic transmission is important in cryptosporidiosis epidemiology in Ethiopia. In addition, they confirm that different *Cryptosporidium* species and subtypes are linked to different clinical manifestations.

## Author Summary

The disease burden of *Cryptosporidium parvum* and role of zoonotic transmission in cryptosporidiosis epidemiology are poorly understood in developing countries. In this study, we examined the distribution and clinical manifestations of *Cryptosporidium* species and subtypes in HIV/AIDS patients in Addis Ababa, Ethiopia. Using molecular diagnostic tools, we detected *Cryptosporidium* infection in 26.9% of 520 HIV/AIDS patients studied. We have shown a very high diversity of *Cryptosporidium* species and subtypes in these patients, but unlike in other developing countries, *C. parvum* is overwhelmingly the dominant species in the study community, responsible for ∼65% *Cryptosporidium* infections. The common occurrence of *C. parvum* zoonotic subtype family IIa, combined with calf contact as a significant risk factor, suggest that zoonotic transmission is important in cryptosporidiosis epidemiology in HIV/AIDS patients in Ethiopia. We have also shown that different *Cryptosporidium* species and subtypes are linked to different clinical manifestations. Improved hygiene and avoidance of calf contact should be advocated to reduce cryptosporidiosis transmission in HIV/AIDS patients in the study setting.

## Introduction

*Cryptosporidium* is an important protozoan parasite affecting HIV/AIDS patients, causing diarrhea, wasting syndrome, and reduced life quality [1]. Since specific therapy or vaccine for the control of this parasite is not yet available, preventing infections depends on avoiding exposure to the parasite and maintaining immune competence. In industrialized nations, access to highly active antiretroviral therapy (HAART) has significantly reduced the morbidity and mortality by cryptosporidiosis [2]–[4]. Nonetheless, cryptosporidiosis is still a major threat to AIDS patients who do not have access to HAART, especially in developing countries [5]–[8]. In industrialized nations, transmission of cryptosporidiosis via contaminated drinking and recreational water and contact with infected farm animals remains a major public health problem in both HIV-positive and immunocompetent persons [9]–[11].

The use of molecular epidemiologic tools has provided new insights into the diversity of *Cryptosporidium* species infecting humans and animals [12]. So far, 26 *Cryptosporidium* species have been described [13]–[17]. Most human cases are caused by *C. hominis* and *C. parvum* [12]. The latter also infects some other mammals, notably calves and lambs, and is responsible for most zoonotic infections in humans. Several other *Cryptosporidium* species are seen in humans at lower frequency, including *C. meleagridis*, *C. felis*, *C. canis*, *C. ubiquitum*, and *C. cuniculus* [12]. More recently, a new species, *C. viatorum*, has been described in 10 travelers returning to Great Britain from the Indian subcontinent [17]. This species appears to be a human-specific pathogen and has since been found in 2 Swedish travelers to Africa and Latin America [18]. In children and HIV-positive persons, differences in clinical manifestations have been observed among different *Cryptosporidium* species, especially between *C. hominis* and *C. parvum*, with the former more virulent than the latter [19]–[21]. In addition, infections with *C. parvum* were associated with chronic diarrhea and vomiting in HIV-positive persons more frequently than was *C. hominis* [21].

Sequence characterization of the 60-kDa glycoprotein (gp60) gene has been commonly used in subtyping *C. hominis* and *C. parvum* [12]. Differences have been observed in host specificity among *C. parvum* subtype families and in virulence among *C. hominis* subtype families. Thus, *C. parvum* subtype family IIa is commonly found in calves, IId is mostly found in lambs and goat kids, whereas IIc is mostly found in humans. Within *C. hominis*, studies in Peru have shown that subtype family Ib was more virulent than other subtype families in children, whereas subtype family Id was more virulent than subtype families Ia and Ie in HIV-positive patients [20], [21].

Cryptosporidiosis is endemic in Ethiopia; occurrence rates of 7.6% to 43.6% were reported in HIV/AIDS patients [22]–[26]. Some potential risk factors for cryptosporidiosis occurrence included contamination of drinking water, contact with calves, living in overcrowded households with many family members, and poor personal hygiene. Thus far, only one study has genetically characterized *Cryptosporidium* spp. from Ethiopia. In the study, 39 of the 41 specimens genotyped had *C. parvum*, one had *C. hominis*, and one had both species. All 12 *C. parvum* specimens subtyped by sequence analysis of the gp60 gene belonged to the subtype family IIa [22].

In the present study, we examined the occurrence of *Cryptosporidium* infection in HIV/AIDS patients in Ethiopia and characterized *Cryptosporidium* spp. at the species, subtype family, and subtype levels. We also examined the association between clinical manifestations and infections with specific *Cryptosporidium* species and subtype families. Data generated from the study have clearly shown a dominance of *C. parvum* in the study population, importance of zoonotic transmission in the epidemiology of cryptosporidiosis in Ethiopia, and differences in clinical manifestations among *Cryptosporidium* species and subtypes.

## Materials and Methods

### Ethical statement

The research protocol was approved by the Ethical Clearance Committee of the Addis Ababa University. All study participants had given written informed consent before enrollment into the study. When the study participant was a child, written consent was obtained from his or her parent or guardian. Researchers at the Centers for Disease Control and Prevention (CDC) had no contact with patients and no access to personal identifiers. Laboratory work on the study specimens was covered under CDC IRB protocol No. 990115: “Use of residual human specimens for the determination of frequency of genotypes or sub-types of pathogenic parasites”.

### Study population and enrollment

This study was cross-sectional in nature. It was conducted between September 2009 and December 2011 in Addis Ababa, Ethiopia. A total of 520 HIV/AIDS patients were recruited from in-patients (hospitalized) and outpatients attending the Tikur Anbessa Hospital, Addis Ababa University, and patients referred to the study by attending physicians. The criteria for inclusion in the study were documented HIV infection, the ability to provide informed consent by the patient or the guardian, and willingness to provide one stool specimen. A structured questionnaire was used to collect CD4+ cell counts, demographic data, clinical symptoms, HAART history, antibiotics usage, and animal exposure history. Each participant was asked to provide a single fresh stool specimen.

### DNA extraction

Stool specimens were stored in 2.5% potassium dichromate at 4°C and shipped to the CDC laboratory in Atlanta for screening and molecular characterization of *Cryptosporidium* spp. by PCR. After washing the stool specimens twice with distilled water, genomic DNA was extracted from 0.5 ml of fecal materials using a FastDNA SPIN Kit for Soil (MP Biomedicals, Solon. OH) and eluted in 100 µl of reagent-grade water following the manufacturer-recommended procedures. DNA was stored at −80°C until analyzed by PCR.

### *Cryptosporidium* species detection, genotyping and subtyping

*Cryptosporidium* oocysts present in the specimens were detected by nested PCR amplification of an approximate 830 bp fragment of the small subunit (SSU) rRNA gene as described previously [27]. *Cryptosporidium* species were determined by restriction fragment length polymorphism (RFLP) analysis of the secondary PCR products using endonucleases *Ssp*I and *Vsp*I [27]. PCR products and restriction fragments were subjected to electrophoresis in 1.5% and 2% agarose gels, respectively, and visualized after staining with GelRed (Biotium Inc., Hayward, CA). All secondary PCR products from species other than *C. parvum* were sequenced to confirm the identification. Specimens that contained *C. parvum* or *C. hominis* were further subtyped by DNA sequencing of the nested PCR product of the gp60 gene [28]. Each specimen was analyzed at least twice by PCR at each locus using 2 µl of the DNA extraction per PCR. As a positive control, *C. baileyi* DNA was used in SSU rRNA PCR and *C. hominis* DNA was used in gp60 PCR. A negative control using DNase-free water was also included in each PCR run.

### Sequence analysis

PCR products were sequenced using the forward and reverse primers of the secondary PCR. An intermediary sequencing primer gp60-R3 [5′-GAG ATA TAT CTT GTT GCG-3′] was also used in the sequencing of gp60 PCR products. DNA sequencing was done using the ABI BigDye Terminator v. 3.1 Cycle Sequencing Kit (Applied Biosystems, Foster City, CA, USA) and an ABI3130 Genetic Analyzer (Applied Biosystems). Sequence accuracy was confirmed by sequencing of two PCR products from each positive specimen. Nucleotide sequences obtained were aligned with reference sequences using the ClustalX 1.81 package (http://www.clustal.org/) to identify *Cryptosporidium* species and *C. parvum* subtypes. Subtypes of *C. parvum* and *C. hominis* were named based on the established nomenclature system [29]. Unique sequences generated in this study were deposited in GenBank under accession numbers AB830575 to AB830590.

### Statistical analysis

Data from the study were analyzed using the SPSS 20.0 for Windows software (IBM Corp, Armonk, NY) at three levels of parasite categorization: presence of *Cryptosporidium*, species of *Cryptosporidium*, and subtype families of *C. parvum* and *C. hominis*. Data from persons infected with low-frequency species were pooled based on their genetic similarities. Univariate and multivariate logistic regression modeling were used to analyze the association between *Cryptosporidium* infection and clinical symptoms or risk-factors while adjusting for potential confounders when the sample size was sufficient. For comparisons at the species or subtype family level, separate models and different subsets of the main dataset were run to examine the effect of each species or subtype family, with *Cryptosporidium*-negative as the referent. The Hosmer-Lemeshow test was used to assess the goodness-of-fit of each multivariate logistic regression model. The strength of the associations was estimated by odds ratios (OR) and 95% confidence intervals (CI). The association was considered statistically significant when the *P* value obtained was smaller than 0.05.

## Results

### Study patients and *Cryptosporidium* occurrence

Among the 520 HIV/AIDS patients who participated in this study, 276 (53.1%) were males and 244 (46.9%) were females. The median age of the study participants was 41 years (range: 7 months to 86 years), and the mean CD4+ cell count was 278 cells/µL. Almost one-third (32.9%) of the study patients were bedridden and hospitalized. Of the 520 stool specimens examined in this study, 140 (26.9%) were positive for *Cryptosporidium* by the SSU rRNA-based PCR technique (Table 1). There were no significant differences in prevalence of *Cryptosporidium* infection among age groups and between the male and female gender (Table 1). No significant association between hospitalization and *Cryptosporidium* infection was found; percentages of *Cryptosporidium* infection in hospitalized and non-hospitalized patients were 35.7% and 37.4% (*P* = 0.83). In addition, no significant difference was observed in the occurrence of vomiting, diarrhea, age, HAART, antibiotic use, animal contact and CD4 between inpatients and outpatients. There was also no association between infections with *Cryptosporidium* spp. or any specific species and hospitalization rates (data not shown).

**Table 1:** Occurrence of *Cryptosporidium* spp. (n = 140 cases) in 520 HIV/AIDS patients in Ethiopia by age and gender*.

Age group (year)	Sample size (%)			No. of patients infected with *Cryptosporidium* (%)			No. of patients infected with each species															
	M	F	Total	M	F	Total	*C. parvum*		*C. hominis*		*C. viatorum*		*C. meleagridis*		*C. felis*		*C. canis*		*C. xiaoi*		*C. parvum+C. hominis*	
							M	F	M	F	M	F	M	F	M	F	M	F	M	F	M	F
<5	157 (50.5)	154 (49.5)	311 (59.8)	47 (29.9)	35 (22.7)	82 (26.4)	26	22	11	6	4	3	2	0	1	3	2	0	1	0	0	1
6–10	65 (62.5)	39 (37.5)	104 (20)	19 (29.2)	8 (20.5)	27 (26.0)	13	5	3	1	1	1	1	0	0	1	0	0	1	0	0	0
11–15	22 (86.6)	4 (13.4)	26 (5)	7 (31.8)	1 (25.0)	8 (30.8)	5	1	2	0	0	0	0	0	0	0	0	0	0	0	0	0
>15	32 (42.7)	43 (57.3)	75 (14.4)	9 (28.1)	13 (30.2)	22 (29.3)	7	12	1	1	1	0	0	0	0	0	0	0	0	0	0	0
Unknown	0	4	4 (0.8)	0	1 (25.0)	1 (25.0)	0	1	0	0	0	0	0	0	0	0	0	0	0	0	0	0
Total	276 (53.1)	244 (46.9)	520	82 (29.7)	58 (23.7)	140 (26.9)	51	41	17	8	6	4	3	0	1	4	2	0	2	0	0	1

*M: male; F: female.

### *Cryptosporidium* species and *C. hominis* and *C. parvum* subtypes

Species determination by RFLP was successful for 128/140 *Cryptosporidium*-positive specimens (Table 2). *Cryptosporidium parvum* (n = 92) and *C. hominis* (n = 25) were the species most frequently detected, followed by *C. felis* (n = 5), *C. meleagridis* (n = 3), *C. canis* (n = 2), and *C. parvum* and *C. hominis* co-infection (n = 1). All SSU rRNA PCR products from non-*C. parvum* specimens were sequenced, confirming the RFLP results. In addition, 12 specimens demonstrated a RFLP pattern that was similar to *C. parvum*, but with a slightly smaller upper *Ssp*I band. DNA sequences from two of the specimens were identical to a reference sequence of *C. xiaoi* (GenBank accession no. JQ413443) and those from 10 specimens were identical to a reference sequence (GenBank accession no. HM485434) of *C. viatorum*, a recently described species in humans [17].

**Table 2:** Distribution of *Cryptosporidium* species and *C. parvum* and *C. hominis* subtypes in HIV/AIDS patients in Ethiopia (n = 140).

Species	Subtype family	Subtype	No. of patients infected
***C. parvum***			92 (82 subtyped)
	IIa		71
		IIaA13G2R1	1
		IIaA14G2R1	1
		IIaA15G2R1	60
		IIaA16G2R1	1
		IIaA16G3R1	4
		IIaA17G2R1	2
		IIaA18G2R1	1
		IIaA19G1R1	1
	IIb		
		IIbA12	1
	IIc		
		IIcA5G3a	2
	IId		5
		IIdA17G1	1
		IIdA19G1	1
		IIdA22G1	2
		IIdA24G1	1
	IIe		
		IIeA12G1	1
	If-like		
		If-like	2
***C. hominis***			25 (19 subtyped)
	Id		13
		IdA20	10
		IdA24	1
		IdA26	2
	Ib		
		IbA10G2	1
	Ie		
		IeA11G3T3	5
***C. viatorum***			10
***C. felis***			5
***C. meleagridi*** **s**			3
***C. canis***			2
***C. xiaoi***			2
***C. parvum + C. hominis***			1

Subtype family data were obtained from 82 (89.1%) of the 92 participants with *C. parvum* and showed the presence of subtype families IIa, IIb, IIc, IId, IIe, and a new subtype family genetically related to If in 71, 1, 2, 5, 1, and 2 persons, respectively. In contrast, 19 (76%) of the 25 participants with *C. hominis* were successfully subtyped and showed the presence of subtype families Ib, Id, and Ie in 1, 13, and 5 persons, respectively. Within *C. parvum*, 8 subtypes were from the subtype family IIa. This was followed by subtype families IId with 4, If-like with 2 subtypes, and IIb, IIc, and IIe with 1 subtype each. Thus, participants in the study were infected with 14 subtypes of *C. parvum*. The most frequently detected subtype was IIaA15G2R1, which was seen in 60 patients. Five subtypes were identified in *C. hominis*, including IdA20, IdA26, IdA24, IeA11G3T3, and IbA10G2 in 10, 2, 1, 5, and 1 patient, respectively (Table 2).

### *Cryptosporidium* species/subtypes and clinical manifestations

*Cryptosporidium* infection was significantly associated with diarrhea in univariate analysis (*P*<0.001), especially in patients with *C. parvum* and *C. hominis* (*P*<0.001 and *P* = 0.012 respectively). Vomiting was also more often seen in *Cryptosporidium*-positive than *Cryptosporidium*-negative patients (63.6% versus 51.8%; *P* = 0.018). Patients with *C. hominis* or rare species (*C. meleagridis/C. felis/C. canis* and *C. xiaoi*) were more likely to have vomiting compared with *Cryptosporidium*-negative patients (*P* = 0.057 and *P* = 0.027, respectively; Table 3).

**Table 3:** Association with diarrhea and vomiting by *Cryptosporidium* species and of *C. parvum* and *C. hominis* subtype in HIV/AIDS patients in Ethiopia.

	Parameter	Total patients	Patients with symptom (%)	Crude		Adjusted#		Goodness of fit##
				OR (95% CI)*	*P-value*	OR (95% CI)	*P-value*	*P*-value
a	**Risk of diarrhea by ** ***Cryptosporidium*** ** positivity**							
	*Cryptosporidium*	140	115 (82.1)	3.033 (1.88–4.90)	0.000	3.120 (1.91–5.10)	0.000	0.93
	No *Cryptosporidium*	380	229 (60.3)	Referent		-	-	-
b	**Risk of diarrhea by ** ***Cryptosporidium*** ** species** **							
	*C. parvum*	92	74 (80.4)	2.711 (1.56–4.72)	0.000	2.829 (1.60–5.02)	0.000	0.98
	*C. hominis*	25	22 (88)	2.199 (1.19–4.05)	0.012	na	na	na
	*C. viatorum*	10	8 (80)	1.382 (0.82–2.33)	0.224	na	na	na
	*C. meleagridis/C. felis/C. canis/C. xiaoi*	12	10 (83.3)	1.347 (0.92–1.98)	0.127	na	na	na
	No *Cryptosporidium*	380	229 (60.3)	Referent		-	-	-
c	**Risk of diarrhea by subtype family** **							
	***C. parvum***							
	IIa	71	56 (78.9)	2.462 (1.34–4.51)	0.003	2.678 (1.43–5.02)	0.002	0.96
	IIb/IIc/IId/IIe/If-like	12	8 (66.7)	1.318 (0.39–4.46)	0.655	na	na	na
	***C. hominis***							
	Id	13	11 (84.6)	3.627 (0.79–16.59)	0.077	na	na	na
	Ib/Ie	6	5 (83.3)	3.291 (0.38–28.50)	0.252	na	na	na
	No *Cryptosporidium*	380	229(60.3)	Referent		-	-	-
d	**Risk of vomiting by ** ***Cryptosporidium*** ** positivity**							
	*Cryptosporidium*	140	89 (63.6)	1.621 (1.09–2.42)	0.018	1.674 (1.11–2.53)	0.014	0.17
	No *Cryptosporidium*	380	197 (51.8)	Referent		-	-	-
e	**Risk of vomiting by ** ***Cryptosporidium*** ** species** **							
	*C. parvum*	92	52 (56.5)	1.208 (0.76–1.91)	0.420	1.275 (0.79–2.06)	0.319	0.48
	*C. hominis*	25	18 (72)	1.546 (0.99–2.42)	0.057	na	na	na
	*C. viatorum*	10	7 (70)	1.294 (0.82–2.04)	0.268	na	na	na
	*C. meleagridis/C. felis/C. canis/C. xiaoi*	12	11 (91.7)	1.788 (1.07–2.99)	0.027	na	na	na
	No *Cryptosporidium*	380	197 (51.8)	Referent		-	-	-
f	**Risk of vomiting by subtype family** **							
	*C. parvum*							
	IIa	71	38 (53.5)	1.070 (0.64–1.78)	0.795	1.065 (0.63–1.80)	0.816	0.33
	IIb/IIc/IId/IIe/If-like	12	7 (58.3)	1.301 (0.41–4.17)	0.658	na	na	na
	*C. hominis*							
	Id	13	9 (69.2)	2.090 (0.63–6.90)	0.217	na	na	na
	Ib/Ie	6	5 (83.3)	4.645 (0.54–40.13)	0.126	na	na	na
	No *Cryptosporidium*	380	197 (51.8)	Referent		-	-	-

# Multivariable logistic regressions were performed to adjust the potential confounders including age, gender, HAART, CD4, and type of patients when sample size was sufficient. na: sample size was too small for multivariate logistic regression analysis.

## Hosmer and Lemeshow test was applied to test the goodness of fit of multivariate logistics regression models.

*95% CI: 95% confidence intervals.

**For each *Cryptosporidium* species or subtype family, patients with the species or subtype family were taken as “positive”, patients who were not infected at all were taken as “negative” (referent), while patients infected with other species or subtype families were not included in this specific model.

Diarrhea was significantly associated with infections with C. *parvum* subtype family IIa compared with *Cryptosporidium*-negative patients (78.9%; P = 0.003). However, there were no significant differences in diarrhea occurrence between patients infected with *C. hominis* subtype families and *Cryptosporidium*-negative patients. Patients infected with different *C. parvum* and *C. hominis* subtype families also did not show significant differences in the occurrence of vomiting compared to *Cryptosporidium*-negative patients (*P*>0.05; Table 3).

Multivariable modeling was attempted for diarrhea and vomiting occurrence in order to adjust for age, gender, type of patients and clinical parameters (HAART and CD4+); however, it did not reveal major differences compared with the crude OR (Table 3).

### *Cryptosporidium* species/subtypes and risk factors

After controlling for other potential risk factors, history of any contact with animals was associated with overall *Cryptosporidium* infections (OR = 1.6; *P* = 0.04), and with *C. parvum* (OR = 2.5; *P* = 0.002) and its subtype family IIa (OR = 2.1; *P* = 0.02) in particular. This association was mostly due to contact with calves (OR = 1.6 and *P* = 0.02 for overall *Cryptosporidium* infection; OR = 1.8 and *P* = 0.01 for *C. parvum*; and OR = 2.0 and *P* = 0.01 for *C. parvum* subtype family IIa; Table 4). However, there was no significant association between *Cryptosporidium* infection and age, gender, HAART history, CD4+ cell counts, antibiotics use, or type of patients (*P* = 0.56, 0.13, 0.59, 0.90, 0.84, and 0.83, respectively). No significant association was found between *Cryptosporidium* infection and CD4+ cell counts or HAART history at the species and subtype levels (Tables S1 and S2).

**Table 4:** Association between animal contact and infection with *Cryptosporidium* species or *C. parvum* and *C. hominis* subtypes in HIV/AIDS patients in Ethiopia*.

Parameter	Total Patients	Patients with animal contact n (%)	Crude		Adjusted#		Goodness of fit##
			OR (95% CI)**	*P-value*	OR (95% CI)	*P-value*	*P-value*
**Animal contact history by ** ***Cryptosporidium***							
*Cryptosporidium*	140	108 (77.1)	1.556 (1.01–2.40)	0.046	1.577 (1.01–2.46)	0.044	0.81
No *Cryptosporidium*	380	247 (65)	Referent		-	-	-
**Animal contact history by ** ***Cryptosporidium*** ** species** ###							
*C. parvum*	92	75 (81.5)	2.376 (1.35–4.19)	0.003	2.495 (1.39–4.48)	0.002	0.49
*C. hominis*	25	21 (84)	1.681 (0.98–2.90)	0.062	na	na	na
*C. viatorum*	10	6 (60)	0.931 (0.61–1.43)	0.744	na	na	na
*C. meleagridis/C. felis/C. canis/C. xiaoi*	12	5 (41.7)	0.788 (0.59–1.055)	0.108	na	na	na
No *Cryptosporidium*	380	247 (65)	Referent		-	-	-
**Animal contact history by subtype family** ###							
*C. parvum*							
IIa	71	57 (80.3)	2.192 (1.18–4.08)	0.012	2.124 (1.13–3.98)	0.019	0.55
IIb/IIc/IId/IIe/If-like	12	9 (75)	1.615 (0.43–6.07)	0.474	na	na	na
*C. hominis*							
Id	13	12 (92.3)	6.462 (0.83–50.24)	0.081	na	na	na
Ib/Ie	6	4 (66.7)	1.077 (0.19–5.96)	0.729	na	na	na
No *Cryptosporidium*	380	247 (65)	Referent		-	-	-
**Calf contact history by ** ***Cryptosporidium***							
*Cryptosporidium*	140	56 (40)	1.679 (1.12–2.52)	0.012	1.628 (1.08–2.46)	0.021	0.55
No *Cryptosporidium*	380	108 (28.4)	Referent		-	-	-
**Calf contact history by ** ***Cryptosporidium*** ** species** ###							
*C. parvum*	92	40 (43.5)	1.937 (1.22–3.10)	0.006	1.839 (1.14–2.97)	0.013	0.69
*C. hominis*	25	15 (60)	1.944 (1.29–2.95)	0.002	na	na	na
*C. viatorum*	10	1 (10)	0.654 (0.33–1.31)	0.230	na	na	na
*C. meleagridis/C. felis/C. canis/C. xiaoi*	12	0	na	na	na	na	na
No *Cryptosporidium*	380	108 (28.4)	Referent		-	-	-
**Calf contact history by subtype family** ###							
*C. parvum*							
IIa	71	33 (46.5)	2.187 (1.30–3.67)	0.003	2.001(1.18–3.40)	0.010	0.98
IIb/IIc/IId/IIe/If-like	12	3 (25)	0.839 (0.22–3.16)	0.796	na	na	na
*C. hominis*							
Id	13	9 (69.2)	5.667 (1.71–18.79)	0.002	na	na	na
Ib/Ie	6	1 (16.7)	0.504 (0.06–4.36)	0.526	na	na	na
No *Cryptosporidium*	380	108 (28.4)	Referent		-	-	-

*Contacts with specific animal species other than cattle were not significant risk factors in this study.

**95% CI: 95% confidence intervals.

# Multivariable logistic regressions were performed to adjust the potential confounders including age, gender, HAART, CD4, and type of patients when sample size was sufficient. na: sample size was too small for multivariate logistic regression analysis.

## Hosmer and Lemeshow test was applied to test the goodness of fit of multivariate logistics regression models.

### For each *Cryptosporidium* species or subtype family, patients with the species or subtype family were taken as “positive”, patients who were not infected at all were taken as “negative” (referent), while patients infected with other species or subtype families were not included in this specific model.

## Discussion

The present findings showed that (1) Ethiopian HIV/AIDS patients were infected with a diverse population of *Cryptosporidium* species, including the unusual species *C. viatorum* and *C. xiaoi*; (2) *C. parvum* was the most frequently detected species; and (3) *Cryptosporidium* species or subtype families were associated with different clinical manifestations. The dominance of *C. parvum* in this study is in agreement with the previous observation in a small study in Ethiopia [22]. It is, however, in sharp contrast with studies of human cryptosporidiosis in other developing countries where *C. hominis* dominates [12], [30], [31]. In Europe, the two species are almost evenly distributed, with *C. parvum* being more prevalent in some reports [32] and *C. hominis* in others [33]. Data from this study also indicate that the recently established species *C. viatorum* is more widely distributed than believed, and humans can be infected occasionally with the sheep and goat parasite *C. xiaoi*. As expected, the prevalence of cryptosporidiosis in HIV+ patients (26.9%) in this study is substantially higher than in the largely healthy persons (8.7%) in a previous study conducted in the same area [22].

Differences in geographical distribution of *C. parvum* and *C. hominis* are generally considered a reflection of differences in infection sources and transmission routes [12]. *C. hominis* is transmitted almost exclusively among humans, whereas *C. parvum*, especially its IIa subtype family, is more likely transmitted zoonotically. In industrialized nations, *C. parvum* infections have often been linked to contact with farm animals, and *C. hominis* infections to contact with children with diarrhea [34]. Both species have been associated with drinking-water outbreaks [33]. The predominance of *C. hominis* in most developing countries suggests that anthroponotic transmission is more important than zoonotic transmission in cryptosporidiosis epidemiology in developing countries in general [12]. In contrast, the dominance of *C. parvum* IIa subtype family in Ethiopia in HIV/AIDS patients suggests that unlike in other developing countries, *Cryptosporidium* infection in Ethiopia is mostly transmitted zoonotically. Previously, it was shown that that small numbers of *C. parvum* infections seen in humans in developing countries were mostly caused by the anthroponotic subtype family IIc [12].

Results of the risk factor analysis support the role of zoonotic transmission in cryptosporidiosis epidemiology in HIV/AIDS patients in Ethiopia. Even though the present study was performed in an urban area, more than 50% of patients had contact with animals, as households in the study area usually have farm animals living inside the residence. Thus, in this study, animal contact, especially with calves, was a significant risk factor for *Cryptosporidium*, especially *C. parvum* and its IIa subtype family. The distribution of *C. parvum* subtypes in this study reinforces the likely occurrence of zoonotic transmission, as the majority of *C. parvum* infections were caused by IIa subtypes (71/82 specimens subtyped), especially its IIaA15G2R1 subtype (60/82 specimens subtyped), which is a dominant *C. parvum* subtype in calves around the world [12]. In contrast, the anthroponotic *C. parvum* subtype family IIc was seen in only 2/82 of *C. parvum* infections.

In addition to IIa, *C. parvum* subtype family IId was also identified in five Ethiopian HIV/AIDS patients. Subtype family IId of *C. parvum* is generally considered a sheep and goat parasite [35], but has been found at high frequency in calves in China, Egypt, and Sweden [36]–[38]. In this study, only one patient infected with IId had contact with sheep in this study. Genotyping and subtyping studies of domestic animals from the study area and additional case-control-studies are needed to support the conclusion on the importance of zoonotic transmission in cryptosporidiosis epidemiology in Ethiopia.

In agreement with previous observations elsewhere [19]–[21], data from the study suggest that different *Cryptosporidium* species and subtypes are linked to different manifestations of cryptosporidiosis. As expected, cryptosporidiosis in our study was significantly associated with the occurrence of diarrhea. This association, however, was largely attributable to *C. parvum* and *C. hominis*; other species, including the newly described *C. viatorum*, were less pathogenic than these two species. Likewise, the significant association between *Cryptosporidium* infection and the occurrence of vomiting was also largely attributable to *C. hominis* and some less frequent species (*C. meleagridis*, *C. canis*, *C. felis* and *C. xiaoi*); *C. parvum* and *C. viatorum* were largely not associated with vomiting. Within *C. parvum*, the IIa subtype family also appeared to be more associated with the occurrence of diarrhea than other *C. parvum* subtype families. We did not observe any effect of CD4+ cell counts and HAART on the occurrence of cryptosporidiosis in HIV/AIDS patients in this study. This was probably largely the result of severe overall immunodeficiency in the study population, as reflected by the low mean CD4+ cell counts (278 cells/µL) and very high hospitalization rate (32.9%) and occurrence of cryptosporidiosis (26.9%).

In conclusion, Ethiopian HIV/AIDS patients with low CD4+ cell counts had an extremely high occurrence of *Cryptosporidium* infection, even when they were on HAART. Although the majority of cryptosporidiosis cases were caused by *C. parvum*, there was a high diversity of *Cryptosporidium* species, with a significant number of cases caused by the newly recognized *C. viatorum*. These *Cryptosporidium* spp. and *C. parvum* subtypes were linked to different clinical manifestations. Therefore, improved hygiene and avoidance of calf contact among this population should be advocated to reduce the occurrence of *Cryptosporidium* infections, especially those caused by *C. parvum* IIa subtypes of calves.

## Supporting Information

Checklist S1 STROBE checklist.
(PDF)

Table S1 Association between CD4+ cell count and infection with *Cryptosporidium* species or *C. parvum* and *C. hominis* subtypes in HIV/AIDS patients in Ethiopia*.
(DOCX)

Table S2 Association between HAART history and infection with *Cryptosporidium* species or *C. parvum* and *C. hominis* subtypes in HIV/AIDS patients in Ethiopia*.
(DOCX)
