# Supplementary material for: Distribution and Clinical Manifestations of Cryptosporidium Species and Subtypes in HIV/AIDS Patients in Ethiopia
Source: PLoS Negl Trop Dis. 2014 Apr 17;8(4):e2831. doi: 10.1371/journal.pntd.0002831 (PMC3990574; doi:10.1371/journal.pntd.0002831)
Supplement: Table S1 — Association between CD4+ cell count and infection with Cryptosporidium species or C. parvum and C. hominis subtypes in HIV/AIDS patients in Ethiopia*. (DOCX) [file pntd.0002831.s002.docx]

**Table S1.** Association between CD4+ cell count and infection with *Cryptosporidium* species or *C. parvum* and *C. hominis* subtypes in HIV/AIDS patients in Ethiopia*

|  | **Parameter** | **Total patients** | **Number of patients with CD4+ count (cells/µl)** | | **Unadjusted OR (95% CI)**** | ***P*** |
| --- | --- | --- | --- | --- | --- | --- |
|  |  |  | **<200** | **>=200** |  |  |
| a | **Infection with *Cryptosporidium*** | | | | | |
|  | *Positive* | 137 | 35 | 102 | 0.97(0.62 -1.52) | 0.90 |
|  | Negative | 379 | 99 | 280 |  |  |
| b | ***Cryptosporidium* species^#^** | | | | | |
|  | *C. parvum* | 89 | 22 | 67 | 0.93(0.55-1.58) | 0.79 |
|  | *C. hominis* | 25 | 10 | 15 | 1.89(0.82-4.33) | 0.14 |
|  | *C. viatorum* | 10 | 1 | 9 | 0.31(0.04-2.51) | 0.28 |
|  | *C. meleagridis/ C. felis/ C. canis/ C. xiaoi* | 12 | 2 | 10 | 0.57(0.12-2.63) | 0.47 |
|  | No *Cryptosporidium* | 379 | 99 | 280 | Referent |  |
| c | **Subtype family^#^** | | | | | |
|  | *C. parvum* |  |  |  |  |  |
|  | IIa | 69 | 19 | 50 | 1.07(0.60-1.91) | 0.81 |
|  | IIb/IIc/IId/IIe/If-like | 10 | 3 | 7 | 1.21(0.31-4.78) | 0.78 |
|  | *C. hominis* |  |  |  |  |  |
|  | Id | 13 | 5 | 8 | 1.77(0.57-5.53) | 0.33 |
|  | Ib/Ie | 6 | 2 | 4 | 1.41(0.26-7.84) | 0.69 |
|  | No *Cryptosporidium* | 379 | 99 | 280 | Referent |  |

* Three in *Cryptosporidium*-positive group and one in *Cryptosporidium*-negative group had missing CD4+ data.

**95% CI: 95% confidence intervals

*#*For each *Cryptosporidium* species or subtype family, patients with the species or subtype family were taken as “positive”, patients who were not infected at all were taken as “negative” (referent), while patients infected with other species or subtype families were not included in this specific model.
