# Supplementary material for: Distribution and Clinical Manifestations of Cryptosporidium Species and Subtypes in HIV/AIDS Patients in Ethiopia
Source: PLoS Negl Trop Dis. 2014 Apr 17;8(4):e2831. doi: 10.1371/journal.pntd.0002831 (PMC3990574; doi:10.1371/journal.pntd.0002831)
Supplement: Table S2 — Association between HAART history and infection with Cryptosporidium species or C. parvum and C. hominis subtypes in HIV/AIDS patients in Ethiopia*. (DOCX) [file pntd.0002831.s003.docx]

**Table S2.** Association between HAART history and infection with *Cryptosporidium* species or *C. parvum* and *C. hominis* subtypes in HIV/AIDS patients in Ethiopia*

|  | **Parameter** | **Total patients** | **HAART** | | **Unadjusted OR (95% CI)**** | ***P*** |
| --- | --- | --- | --- | --- | --- | --- |
|  |  |  | **Yes** | **No** |  |  |
| a | **Infection with *Cryptosporidium*** | | | | | |
|  | *Cryptosporidium* | 140 | 15 | 125 | 1.28 (0.52 - 3.12) | 0.59 |
|  | No *Cryptosporidium* | 379 | 49 | 330 | Referent |  |
| b | ***Cryptosporidium* species^#^** | | | | | |
|  | *C. parvum* | 92 | 14 | 78 | 1.21 (0.64 - 2.30) | 0.56 |
|  | *C. hominis* | 25 | 1 | 24 | 0.28 (0.04 - 2.12) | 0.22 |
|  | *C. viatorum* | 10 | 0 | 10 | na | na |
|  | *C. meleagridis/ C. felis/ C. canis/ C. xiaoi* | 12 | 0 | 12 | na | na |
|  | No *Cryptosporidium* | 379 | 49 | 330 | Referent |  |
| c | **Subtype family^#^** | | | | |  |
|  | *C. parvum* |  |  | |  |  |
|  | IIa | 71 | 11 | 60 | 1.24 (0.61 - 2.51) | 0.56 |
|  | IIb/IIc/IId/IIe/If-like | 11 | 2 | 9 | 1.50 (0.31 - 7.13) | 0.61 |
|  | *C. hominis* |  |  |  |  |  |
|  | Id | 13 | 0 | 13 | na | na |
|  | Ib/Ie | 6 | 0 | 6 | na | na |
|  | No *Cryptosporidium* | 379 | 49 | 330 | Referent |  |

*One patient in *Cryptosporidium*-negative group had missing HAART history data.

**95% CI: 95% confidence intervals. na: sample size was too small for logistic regression analysis.

#For each *Cryptosporidium* species or subtype family, patients with the species or subtype family were taken as “positive”, patients who were not infected at all were taken as “negative” (referent), while patients infected with other species or subtype families were not included in this specific model.
